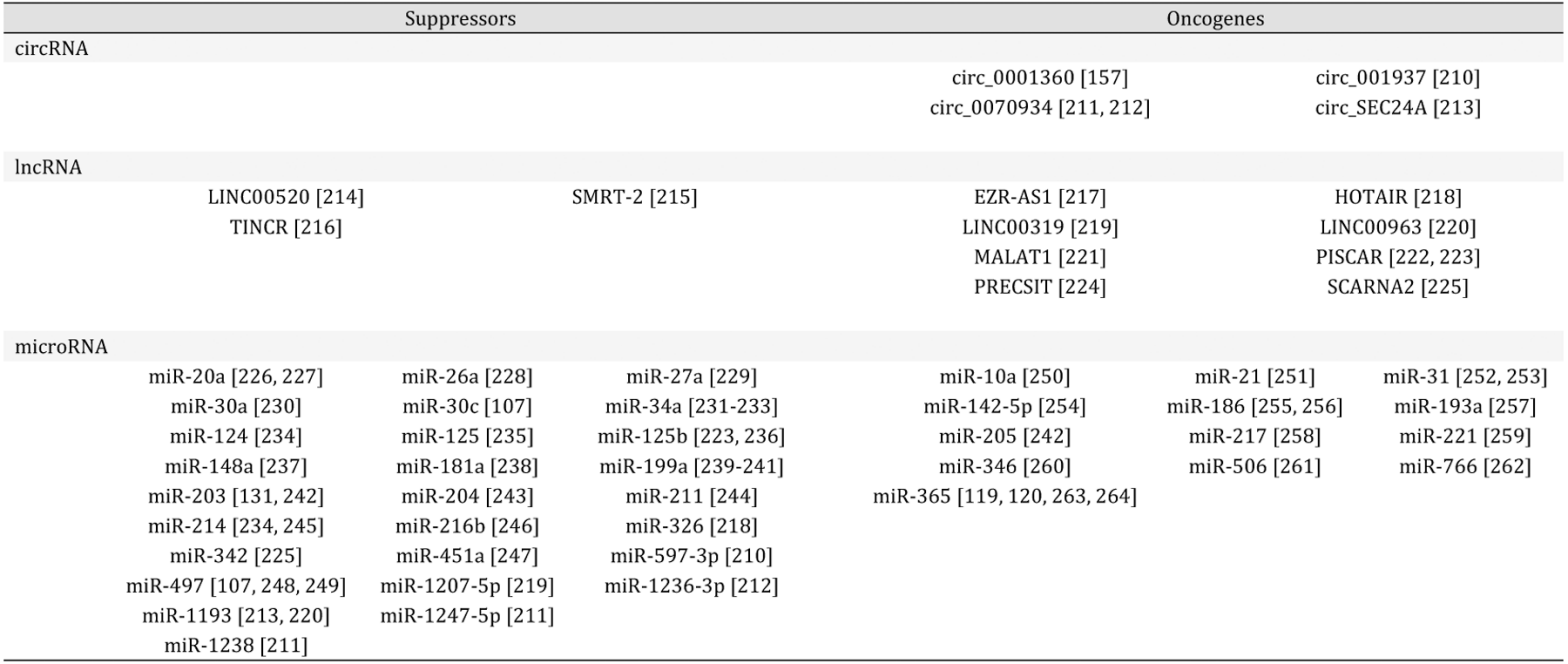# Oh, the Mutations You’ll Acquire! A Systematic Overview of Cutaneous Squamous Cell Carcinoma

**DOI:** 10.33594/000000433

**Published:** 2021-09-22

**Authors:** Stephenie Droll, Xiaomin Bao

**Affiliations:** a Department of Molecular Biosciences, Northwestern University, Evanston, IL, USA,; b Department of Dermatology, Northwestern University, Chicago, IL, USA,; c Robert H. Lurie Comprehensive Cancer Center, Northwestern University, Chicago, IL, USA

**Keywords:** cSCC, Skin Cancer, Keratinocyte Carcinoma, Epidermis

## Abstract

Nearly two million cases of cutaneous squamous cell carcinoma (cSCC) are diagnosed every year in the United States alone. cSCC is notable for both its prevalence and its propensity for invasion and metastasis. For many patients, surgery is curative. However, patients experiencing immunosuppression or recurrent, advanced, and metastatic disease still face limited therapeutic options and significant mortality. cSCC forms after decades of sun exposure and possesses the highest known mutation rate of all cancers. This mutational burden complicates efforts to identify the primary factors driving cSCC initiation and progression, which in turn hinders the development of targeted therapeutics. In this review, we summarize the mutations and alterations that have been observed in patients’ cSCC tumors, affecting signaling pathways, transcriptional regulators, and the microenvironment. We also highlight novel therapeutic opportunities in development and clinical trials.

## Introduction

**Fig. 1.**
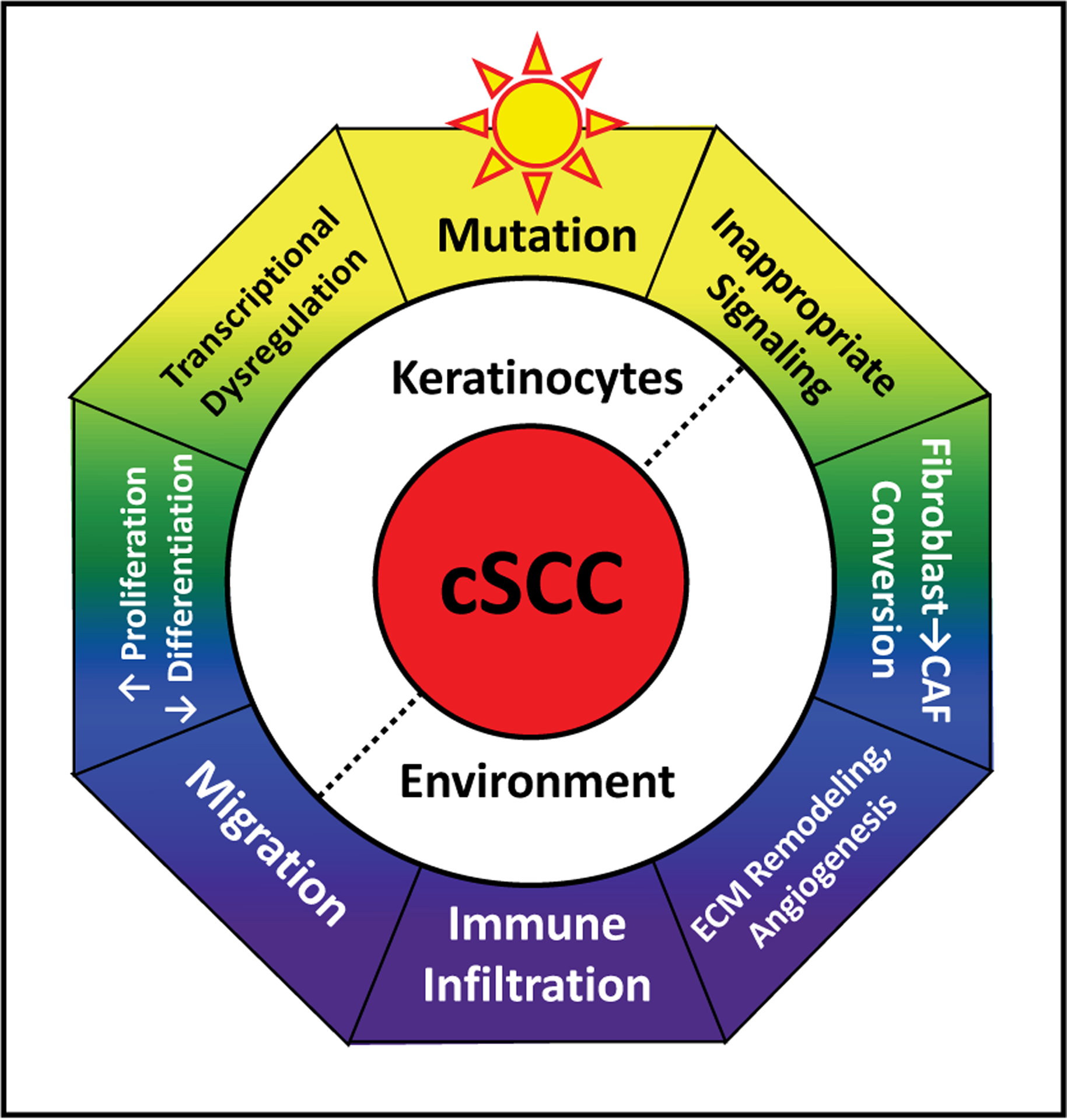
Factors contributing to cSCC initiation and progression. Mutation, usually through exposure to UV radiation, initiates disease. Aberrant signaling and transcriptional dysregulation disrupts both epidermal keratinocyte and dermal fibroblast behavior. Cancer-associated fibroblasts (CAFs) and infiltrating immune cells remodel the extracellular matrix (ECM), promoting cSCC invasion and angiogenesis.

An invasive type of skin cancer, cSCC is the second-most common cancer worldwide. cSCC represents an incredible emotional, physical, and financial burden for patients and the healthcare system. In the USA alone, cSCC hospitalizes 6.2 per 100,000 people with an average stay of 5.8 days and cost of $66,000 [1] and causes an estimated 15,000 annual deaths [2]. This represents twice the melanoma death rate and matches deaths due to brain, esophageal, oral, or ovarian cancers [3]. Alarmingly, the incidence of cSCC continues to rise [4]. While new therapies are being developed to treat advanced cSCC, this life-threatening condition remains challenging to cure. cSCC is a multifaceted disease with numerous factors influencing its initiation and progression. Rather than focusing on a particular gene or pathway, this review will assess the layers of complexity. Cumulatively, this review will discuss the characteristics of cSCC, dysregulation of signaling pathways, alterations to critical nuclear effectors, and the resulting disorganization of the extracellular environment (Fig. 1). Where possible, we will highlight interactions between these modules, approved drugs, and novel therapeutic targets.

## Characteristics of cSCC

**Fig. 2.**
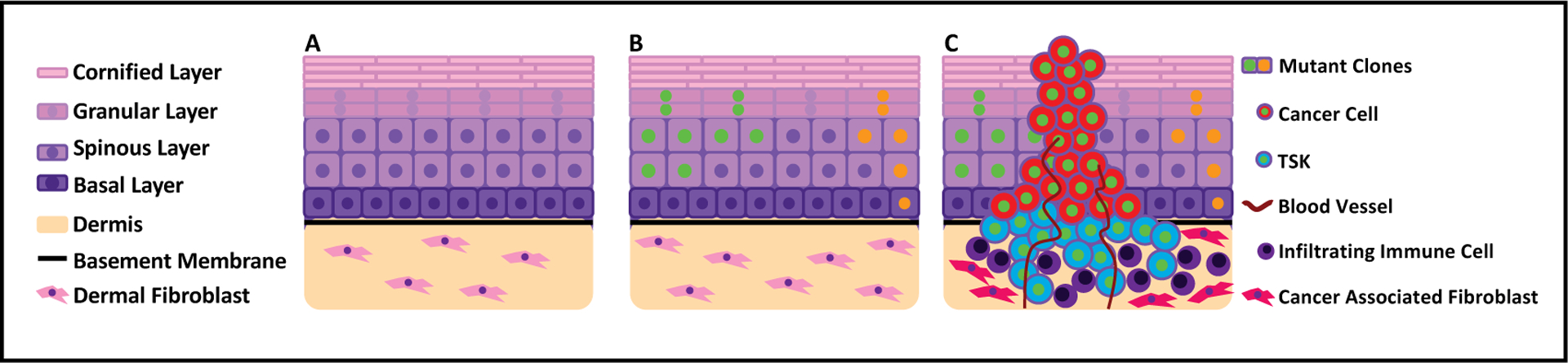
Malignant transformation of the epidermis. A. Normal skin where the dermis and epidermis are separated by an intact basement membrane. Epidermis contains proliferating basal and differentiating spinous, granular, and cornified layers. B. Primarily due to UV radiation, visibly normal skin accumulates mutations, forming dynamically evolving, clonal, mutant populations. C. Invasive cSCC demonstrates full-thickness dysplasia, disruption of the basement membrane, invasion into the dermis, and vascularization. Infiltrating immune cells surround the tumor, and fibroblasts near the tumor adopt a pro-cancerous phenotype. While many tumor cells mimic normal keratinocyte populations, a unique population of tumor-specific keratinocytes forms.

cSCC develops from skin epidermis, a critical physical and immune barrier protecting the human body. Epidermis is composed primarily of keratinocytes organized into multiple layers (termed stratified squamous epithelium). In homeostatic epidermis, keratinocyte proliferation is restricted to the basal layer, which is anchored to the basement membrane, a mechanical barrier of extracellular proteins which separate the epidermal and dermal components of skin. During differentiation, keratinocytes migrate to the upper layers (spinous, granular, and cornified strata), withdraw from the cell cycle, and sequentially activate genes allowing the production of proteins and lipids that contribute to barrier function (Fig. 2a) [5]. Dysregulation of keratinocyte proliferation and differentiation is fundamental to cSCC initiation and progression.

cSCC features the highest mutational burden of all cancers, with a median of 45.2 mutations per megabase (Mb) of genomic DNA [6, 7]. These mutations are substantially enriched in C > T or CC > TT substitutions, the characteristic ultraviolet (UV) mutation signature. Thus, UV radiation is considered the chief epidermal carcinogen [2]. Notably, these mutations occur in morphologically normal skin, but at a lower frequency of 2–6 coding mutations per Mb, where they drive dynamic, temporal, and spatial genetic mosaicism (Fig. 2b) [8]. Clones containing cancer-driver mutations (such as TP53, NOTCH, and RAS) undergo strong positive selection and expand in size [9, 10]. Remarkably, the mutational burden accumulated in normal epidermis is greater than that of many solid tumors, yet epidermis forestalls malignant transformation for decades [10]. How epidermis can tolerate such a high level of mutation remains unclear. Yet, this high mutational burden complicates identification of bona fide cSCC drivers and the development of targeted therapeutics, despite 60% of cSCC tumors potentially harboring mutations targetable by an existing small molecule therapy developed for other cancers [11].

The architecture of cSCC tumors is highly complex, composed of keratinocytes and a variety of stromal cells. At the invasive front of cSCC, a population of CD133^+^/CD45^−^ keratinocytes (~1% of cells) act as tumor initiating cells (cancer stem cells). Xenograft of these sorted keratinocytes reliably replicates the histology of the original tumor [12]. Additionally, recent single-cell RNA sequencing of cSCC tumors also identified a unique subpopulation of tumor-specific keratinocytes (TSKs) at the invasive front (Fig. 2c). TSKs demonstrate strong upregulation of genes related to cell motility and extracellular matrix remodeling. Spatial gene-expression analysis identified proximity of TSKs with stromal cells (cancer-associated fibroblasts and vascular cells) as crucial for cancer progression [13]. Thus, TSKs can function as signaling hubs among different cell types in cSCC. In addition to TSKs at the invasive front, cSCC contains subpopulations of keratinocytes similar to normal tissue, but these cells generally exhibit altered metabolic and immune response, decreased apoptosis and differentiation, and epithelial-to-mesenchymal transition (EMT) [13]. Interestingly, cSCC cell lines also possess TSKs after being xenografted to mice, suggesting that TSKs may not necessarily have additional driver mutations compared to the other populations. Rather, the unique gene expression patterns of TSKs are likely to be induced and enhanced by the local environment.

The majority (65–80%) of cSCC develops from premalignant lesions called actinic keratosis (AK) and Bowen’s disease (BD). AK presents as a small, red, scaly patch, and forms when dysplastic keratinocytes proliferate. AK represents the third most common reason for dermatological consultation since the lesions are cosmetically displeasing and sometimes painful. BD tends to be redder, scalier, and slow-growing lesions characterized by full-thickness dysplasia that does not breach the basement membrane [14], sometimes referred to as “cSCC in situ.” While estimates vary, a single AK poses a 10% or 20% risk progressing to cSCC in immunocompetent and immunocompromised patients respectively while a BD lesion poses a 16.3% risk [14–16]. Treatments for AK and BD include: topical drugs (5-fluorouracil, imiquimod, ingenol mebutate, diclofenac), photodynamic therapy, and removal (surgical, cryotherapy, laser) [16]. While treatment reduces lesion size and number, patients are burdened by significant skin irritation, length of treatment, and lesion recurrence. For prophylaxis, oral retinoids likely provide no benefit, but proper sunscreen use reduces AK formation [16]. Oral nicotinamide promotes DNA repair by preventing ATP depletion after UV exposure. This safe and cheap drug reduces AK lesions [17] and cSCC occurrence by 30% in immunocompetent patients [18]. AK and BD represent a precancerous condition, but lesion growth and bleeding may suggest progression to cSCC.

Compared to the immunocompetent population, transplant patients have a dramatically increased (32–198 fold) risk of developing aggressive, relapsing cSCC [19]. While immunosuppression prevents graft rejection, it increases cSCC risk, which then threatens graft viability. In addition to reducing immune surveillance, calcineurin inhibitors and azathioprine, directly contribute to increased mutational burden. Azathioprine photosensitizes skin and increases reactive oxygen species (ROS) production upon UV exposure [20]. Calcineurin inhibitors increase cSCC occurrence by inhibiting the nucleotide and base excision DNA repair pathways [21]. Switching from calcineurin to mTOR inhibition halved skin cancer rates in transplant patients [22], as mTOR inhibitors can also suppress DNA damage, proliferation and angiogenesis [23]. Prevention and treatment of cSCC in immunocompromised patients represents a critical and growing clinical problem. Prophylactic retinoids (vitamin A derivatives) [24] and capecitabine (inhibitor of DNA repair) are currently prescribed off-label for AK and cSCC in transplant patients. Metaanalyses suggest some efficacy but also serious adverse reactions. No controlled clinical trial has evaluated prophylaxis efficacy and safety [25].

Until recently, treatment for advanced cSCC was limited to surgery and off-label therapies that lacked robust evidence of efficacy or safety. Surgical removal is still the frontline treatment for cSCC, but results in a 3–8% recurrence rate, even when the original tumor is properly excised. As cSCC frequently occurs on the face, surgery can be disfiguring. However, tumor placement, advancement, and patient health can prohibit surgical intervention [26]. Therefore, adjuvant radiotherapy is recommended for immunosuppression, incomplete excision, metastasis, perineural invasion, and recurrence [24, 27]. Local recurrence, lymph node metastasis, and distant metastases occur in 3–14%, 3.7–16%, and 5% of patients respectively [28–30]. Risk factors for recurrence and metastasis include: large tumor size, perineural or subcutaneous fat invasion, poor differentiation, and location on the temple, ear, or lip [31].

## Alterations to Signaling Pathways

Cell growth, motility, and survival are strictly controlled by environmental cues in homeostatic tissue. These cues are received by cell surface receptors and processed by different signaling pathways to trigger specific cellular responses. A number of mutations have been identified in distinct components of pathways, bypassing signaling-regulated restrictions to promote uncontrolled growth. To illustrate the complexity of alterations in cSCC, this section will primarily focus on the epidermal growth factor receptor (EGFR) signaling pathway, but will also discuss the roles of NOTCH and TGFβ.

### EGFR Pathway

#### EGFR.

**Fig. 3.**
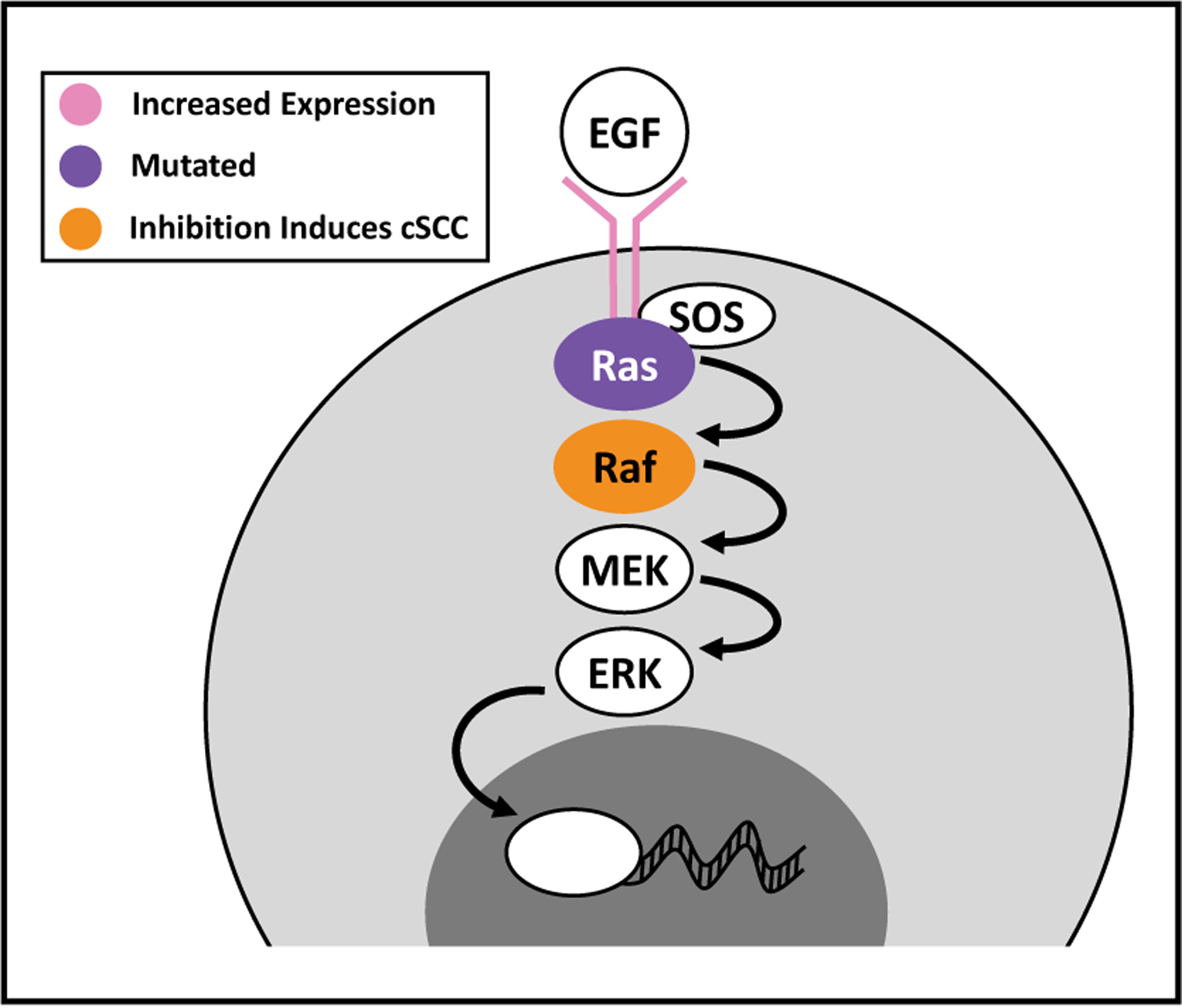
A simplified diagram of the EGFR-MAPK signaling pathway, with highlights of the recurrent pathogenic changes described in patients’ cSCC tumors.

The EGFR pathway plays crucial roles in regulating cell growth and survival. Four genes encode EGFR receptors in the human genome, but only three are expressed by normal keratinocytes: EGFR (ERBB1), HER2 (ERBB2), and HER3 (ERBB3). Seven ligands for EGFR have been identified with varying receptor affinity [32]. Upon activation, EGFR dimerizes and functions as tyrosine kinase to trigger downstream kinase cascades (Fig. 3). Interestingly, UV radiation activates EGFR by preventing its inhibitory dephosphorylation, leading to cyclin D activation and p53 suppression, which induce epidermal hyperplasia. UV radiation can also induce nuclear translocation of EGFR. UV-induced EGFR activation is a unique feature of epidermal tissue [33]. EGFR inhibition reduces UV-induced erythema, hyperplasia, lymphocyte infiltration, cytokine production, and tumor size [34–37].

EGFR copy-number gains occur frequently in cSCC, but also in AK [38], suggesting that the pathway is altered early during cSCC carcinogenesis. Immunohistochemistry (IHC) of cSCC consistently reveals EGFR overexpression (Fig. 3), and aberrant cytoplasmic staining [38–41]. Overexpression independently predicts disease progression and is more prevalent in metastatic than primary tumors [40, 42]. Moreover, a recurrent cSCC-specific EGFR-PPARGC1A fusion was recently identified in 30% of the studied tumors. The fusion protein is constitutively phosphorylated and dimerizes with wild-type EGFR, inducing activation. Cells expressing the EGFR-PPARGC1A fusion form even larger tumors than cells overexpressing EGFR while PPARGC1A overexpression does not induce tumors [43]. In contrast, EGFR mutation is relatively rare, affecting about 4.9% of cSCC cases [44].

Given EGFR’s frequent alteration in cSCC and its critical roles in cancer progression, EGFR represents an attractive therapeutic target with both antibodies and small molecule inhibitors being developed. Cetuximab, a monoclonal antibody targeting EGFR, was approved in 2006 for advanced cSCC of the head and neck. Used alone or with other therapy for unresectable and metastatic cSCC, cetuximab produces an overall response rate around 50% with an acceptable safety profile, even in elderly patients [45–47]. Another EGFR antibody, panitumumab, in combination with the talimogene laherparepvec vaccine (a virusbased melanoma therapy) is currently in phase I trial. On the small-molecule inhibitor front, recent phase II trials of lapatinib and gefitinib produced results only in a subset of patients. Two weeks of lapatinib reduced tumor volume in only 2 of 8 patients. However, two months after treatment, 7 of 8 patients did experience remission of concurrent AK [48]. Gefitinib induced partial response in 6 of 37 and stable disease in 13 of 37 patients with a median duration of 31.4 months [49]. The small-molecule-inhibitor, erlotinib failed to achieve an acceptable response rate in phase II trials [50]. Recently, preclinical screening identified DUBs-IN-3 as a potential cSCC drug. DUBs-IN-3 inhibits USP8, a component of the ubiquitin pathway that shields EGFR and other growth factor receptors from degradation. DUBs-IN-3 treatment reduces EGFR protein and preferentially kills cSCC cells. As DUBs-IN-3 multiple receptors, it may aid in overcoming drug resistance [51].

#### RAS.

EGFR activation leads to phosphorylation and binding of Ras adaptor proteins, docking of guanine nucleotide exchange factors (GEFs), and activation of the Ras GTPase. Activated Ras serves as a signaling hub to activate several downstream kinases. There are three paralogs: H-Ras, K-Ras, and N-Ras. These paralogs are required, but redundant during epidermal development. H-Ras null mice are indistinguishable from wild-type littermates, as K-Ras and N-Ras can compensate for H-Ras in developmental processes [52]. Triple knockout of all three Ras paralogs in epidermal tissue inhibits both proliferation and differentiation, resulting in post-natal lethality. These triple-knockout keratinocytes exhibit G1-arrested senescence, downregulate p63 and c-MYC, and express early, but not late differentiation markers. Constitutive ERK2 activation can rescue proliferation of Ras-null cells and restores c-Myc and p63 expression [53]. These findings highlight the central role of Ras and its downstream signaling in controlling keratinocyte proliferation and differentiation.

Ras mutations affect a small subset of cSCC, with frequencies of: 6–16% H-Ras, 13% K-Ras, and 5% N-Ras (Fig. 3) [7, 44, 54, 55]. Much of the research exploring the role of Ras in cSCC has focused on H-Ras. Expression of H-Ras^G12V^ in keratinocytes causes oncogene-induced senescence [56]. However, in combination with additional oncogenic agents or mutations, Ras can potently drive rapid and aggressive cSCC progression. In the classic DMBA (7,12-dimethylbenz [a]anthracene) plus TPA (12-O-tetradecanoylphorbol-13-acetate) mouse model of cSCC, the mutagen DMBA promotes H-Ras mutation in 95% of tumors while repeated application TPA drives proliferation, leading to hyperplasia. Tumors progressively develop, and metastases form in ≤35% of mice that develop cSCC. Malignancy is influenced by mouse age and genetic background as well as the dose of initiator and the frequency of promoter application [57, 58]. In H-Ras null mice, DMBA/TPA treatment instead triggers K-Ras mutation, which results in 6-fold fewer papillomas but 4-fold more metastasizing cSCC [52, 59]. Although the DMBA/TPA model is invaluable to study cSCC, it must be noted that human skin responds to TPA differently than mouse skin. In another popular cSCC model, H-Ras mutation cooperates with CDK4 overexpression, which promotes G1 escape, to induce invasive cSCC from the xenografted human keratinocytes [60]. Age-associated inflammation promotes H-Ras mutation-driven tumorigenesis. When an inducible, basal, H-Ras^G12V^ construct was induced in young versus old mice, the young mice developed dysplasia while aged mice developed greater dysplasia with hyperplasia, inflammation, and marked macrophage and T-cell infiltration, ultimately developing cSCC. For young mice, dysplasia resolved upon 4-OHT withdrawal while aged epidermis remained hyperplastic and inflamed [61]. These studies highlight the importance of context in carcinogenesis, even when testing the same oncogene.

Although vital for Ras function, the GEFs remain understudied in epidermal development and tumorigenesis. While SOS1 is required for epidermal development, SOS2 is dispensable. SOS1 knockout in mice decreases keratinocyte proliferation, wound healing, and the number of epidermal and dermal layers and delays cSCC formation. In contrast, SOS2 knockout causes no change to skin architecture or tumor formation [62]. RasGRP1 is overexpressed in cSCC, yet RasGRP1 overexpression in normal keratinocytes induces G2 arrest. Like oncogenic H-Ras, RasGRP1 overexpression alone inhibits epidermal stratification in organotypic culture, but cooperates with mutant p53 to transform keratinocytes [63]. The limited evidence suggest that the role of Ras associated proteins in cSCC deserves more thorough investigation.

#### RAF and MEK.

Raf proteins are serine/threonine kinases directly activated by Ras. Three Raf genes are encoded by the human genome: a-Raf, b-Raf, and c-Raf [64]. b-Raf mutations are highly prevalent in melanoma but rare in cSCC. Intriguingly, b-Raf inhibition, used to treat melanoma, induces Ras signaling and cSCC in a subset of patients (Fig. 3) [65, 66]. These cSCC tend to occur in sun-protected skin, and 60% harbor Ras mutation [66]. In the DMBA/TPA mouse model, b-Raf inhibition accelerates tumor formation without increasing tumor number [67]. In the presence of K/N-Ras mutation, b-Raf inhibitors drive b/c-Raf heterodimerization activating c-Raf, MEK, and ERK [67, 68]. In this context, MEK inhibition does not affect established tumors, but suppresses new tumor formation by 91% [67]. These findings reveal complex regulation among the different modules of the Ras pathway.

MEK1 and MEK2 kinases are downstream of Raf. MEK1 and MEK2 are individually dispensable for epidermal development while double knockout induces severe proliferation and barrier defects and rapid post-natal lethality. Downstream ERK2 activation rescues this hypoplasia [69]. Activated Raf can utilize either MEK1 or MEK2 to induce hyperplasia [69]. However, overexpression of either wild-type or kinase dead MEK1, but not MEK2, induces proliferation and reversible epidermal hyperplasia [70], suggesting that MEK1 upregulation differs from MEK2 in contributing to cSCC progression. MEK inhibitors were reported to cause senescence of cSCC cells *in vitro* and abrogate tumor development in UV-induced cSCC mouse models [64]. MEK inhibitors may be a valuable cSCC therapeutic option, but toxicity must still be carefully evaluated.

#### NOTCH Pathway

The NOTCH pathway, essential for both embryonic development and epidermal homeostasis, also plays a crucial role in cSCC progression. Four membrane bound NOTCH receptors are encoded by the human genome but only NOTCH1, 2, and 3 are expressed in keratinocytes. Upon activation by a battery of ligands, the NOTCH intracellular domain translocates to the nucleus to regulate transcription. NOTCH is frequently mutated in cSCC: NOTCH1 42.6% to 75%, NOTCH2 18% to 59%, and NOTCH3 7.4% [7, 44, 55, 71, 72]. While the role of NOTCH in cancer appears to be tissue specific, NOTCH1 generally functions as a tumor suppressor in the epidermis. NOTCH1 knockout induces epidermal hyperplasia, disrupts differentiation, and promotes tumor formation upon chemical induction [73, 74]. At the transcriptional level, MAML1 functions as a critical NOTCH coactivator. A dominant negative mutant of MAML1 inhibits NOTCH signaling and induces epidermal hyperplasia with dermal hypoplasia and lesions consistent with AK and cSCC. This hyperplastic tissue features cyclin D accumulation in the nuclei of suprabasal cells, suggesting suprabasal cells fail to exit the cell cycle during differentiation without NOTCH signaling [75]. Additionally, engineered pulses of NOTCH1 activation rapidly induce spinous differentiation and the DNA damage response, but induction of terminal differentiation is delayed. NOTCH1 activation also fails to downregulate basal markers, such as p63 [76]. Together these results show that NOTCH1 is necessary, but not sufficient for induction of epidermal differentiation. Yet, despite the described role as a differentiation inducer and tumor suppressor, NOTCH1 is overexpressed in CD133+ cells, which can initiate new tumor formation upon mouse xenograft. NOTCH1 silencing reduces the CD133+ population in cSCC cell lines by 40% and colony formation [77], suggesting that NOTCH1 may also play a role in cSCC initiation.

#### TGFβ Pathway

The TGFβ signaling pathway is comprised of 35 TGFβ ligands, 13 receptors, and eight SMAD effectors. For a detailed TGFβ review, see Wu et al. [78]. The role of TGFβ signaling changes as cSCC progresses. In early tumorigenesis, TGFβ signaling generally acts as a suppressor by reducing keratinocyte proliferation, but switches to an oncogenic role as cSCC advances. For example, TGFβ secreted by the stroma, slows cSCC cell cycling at the invasive front of the tumor, which helps to confer resistance to cisplatin chemotherapy. Consequently, these TGFβ responsive cells can induce tumor recurrence [79].

**Fig. 4.**
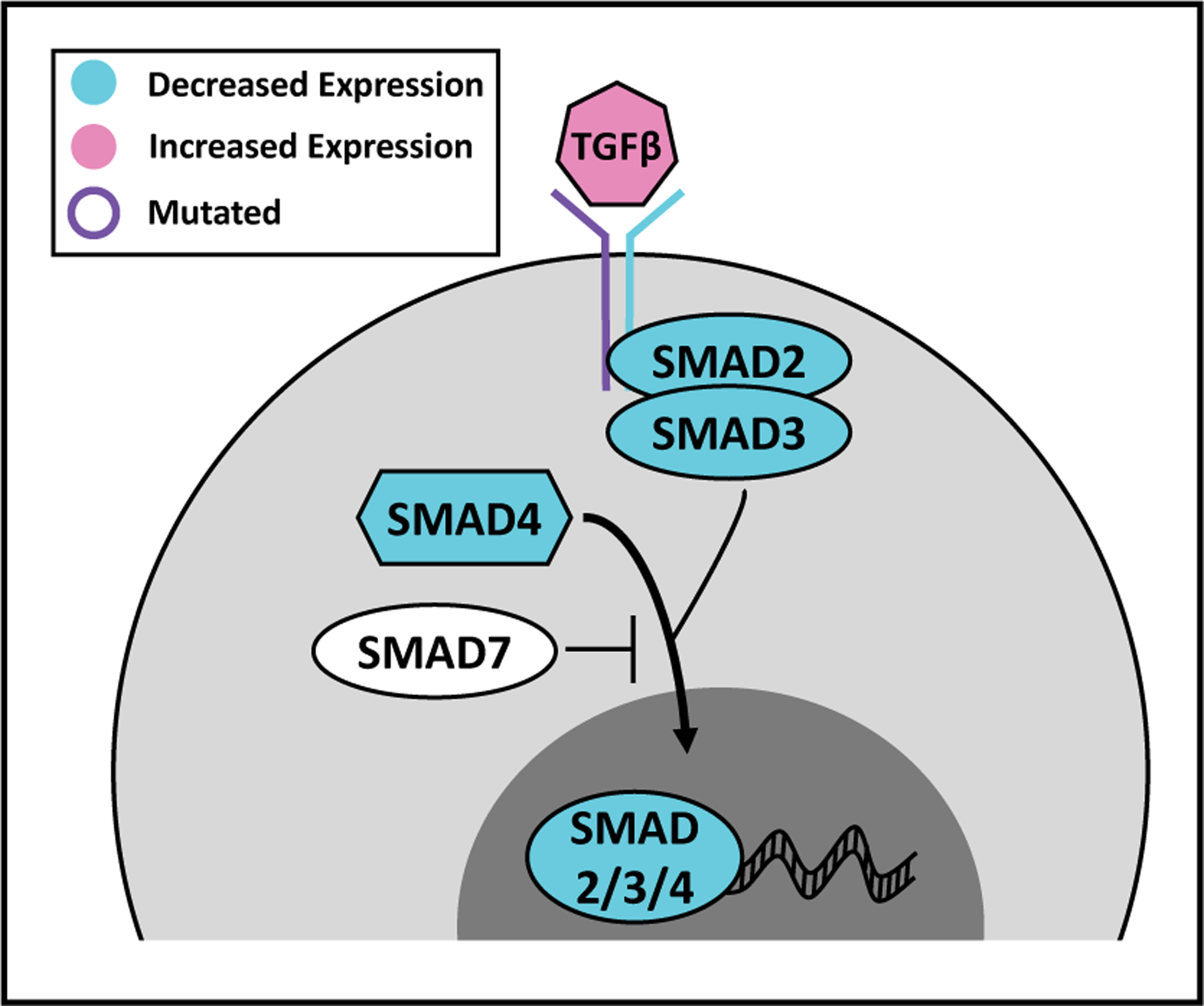
A simplified diagram of the TGFβ signaling pathway, with highlights of the recurrent pathogenic changes described in patients’ cSCC tumors.

TGFβ ligand initiates signaling by activating a receptor complex of TGFBR1 and TGFBR2, which then phosphorylates SMADs 2 and 3. In cSCC, TGFBR1 and TGFBR2 are recurrently mutated (Fig. 4), primarily resulting in loss-of-function and inactivation of SMAD signaling. TGFBR2 mutant cSCC cell lines (SCCIC8, SCCIC12) fail to respond to TGFβ treatment and continue proliferating [80]. On the surface, this would suggest that SMAD signaling suppresses cSCC development, but the downstream SMAD proteins actually play opposing roles. Some studies indicate that SMADs function as suppressors for cSCC. For example, SMAD2 and SMAD4 are recurrently lost in cSCC tumor samples by IHC (Fig. 4). The reduction of phosphorylated SMAD2 and SMAD3 in human cSCC correlates to larger, thicker tumors [81]. Epidermal SMAD4 knockout induces spontaneous cSCC formation in 70% [82] to 100% [83] of mice within a year. Likewise, epidermal SMAD2 knockout promotes cSCC formation during chemical carcinogenesis [84]. In contrast, SMAD3 knockout dramatically reduces the number of papillomas and cSCC tumors that form in response to DMBA/TPA, suggesting that SMAD3 promotes cSCC progression [85]. In addition to regulating proliferation, TGFβ signaling can serve as a powerful monocyte chemoattractant. Mice overexpressing the inhibitory SMAD7 are neither more nor less susceptible to chemical carcinogens [86]. Interestingly both SMAD3 knockout and SMAD7 overexpression result in decreased tumor macrophage infiltration [85]. Altogether, the conflicting roles of the individual components make drugging the TGFβ pathway fraught with the danger of exacerbating cSCC.

## Alterations to Effectors

### p53

The most commonly mutated gene in cSCC is p53 with reported mutation rates of 64–85.2% [7, 44, 54]. p53 functions as the “guardian of the genome,” and suppresses cancer initiation by promoting cell cycle arrest, DNA repair, or apoptosis in response to metabolic disorder and DNA damage. Since p53 functions as a tetramer, the mutant protein can antagonize the remaining wild-type protein and can even abolish its transcription regulatory activity.

Mutant p53 resists protein degradation and accumulates in the nucleus, which enables IHC analyses of clinical samples [87]. IHC demonstrates that p53 mutation occurs early during epidermal carcinogenesis. In >90% of AK, more than half the cells are p53 positive. Positivity significantly correlates with cumulative UV exposure, older age, and high-grade dysplasia [88]. AK that persist demonstrate more intense p53 staining than AK that spontaneously regress [89]. p53 staining intensity further increases upon progression to cSCC, and high positivity delineates poorly differentiated from well-differentiated tumors [90]. In metastatic disease, p53 mutation is retained [71] and promotes aggressive tumor behavior such as recurrence [54], but additional p53 mutations are not usually acquired [91]. These studies demonstrate that p53 mutation promotes cSCC initiation and progression.

In addition to damaging genome stability, p53 mutations can cooperate with other oncogenes, such as Ras, to suppresses differentiation, promote proliferation, and induce large, poorly differentiated tumors that undergo epithelial to mesenchymal transition (EMT) [56]. Therefore, numerous therapeutics targeting p53 have been developed to restore its anti-cancer functions. MDM2 functions as the primary inhibitor of p53 by marking it for proteasomal destruction. Eight inhibitors of the MDM2-p53 interaction are undergoing phase I and II clinical trials. In other solid tumors and leukemias harboring wild-type p53, relieving MDM2 inhibition holds promise for treating malignancy. However, only a minority of cSCC preserve wild-type p53. In addition, small molecules that can fold mutant p53 into the wild-type conformation are being developed, but only two compounds, APR-246 and COTI-2, have reached clinical trials [92, 93]. It remains unclear whether these compounds target specific mutations or can broadly refold mutant p53. Another therapeutic strategy reduces mutant p53 protein accumulation. One such therapy, ganetespib, is a Hsp90 inhibitor that also reduces p53 accumulation, but a phase III clinical trial for advanced non-small cell lung cancer failed to show improved survival [94]. The promise of p53 therapies remains untested in cSCC. However, it will be critical that these p53 drugs, especially the refolding agents, do not also inadvertently stabilize the homologous p63 protein, a key cSCC driver. If so, p53 therapies could instead promote cSCC growth.

### p63

p63 is a p53 paralog and serves as the master regulator of epidermal development and homeostasis. The p63 locus produces different isoforms including TA (has p53-like transactivation domain) and ΔN (lacks transactivation domain). p63 deletion in mice induces a lethal absence of epidermis due to dysregulation of both proliferation and differentiation [95]. ΔNp63α is the predominant functional isoform in the epidermis, where it promotes basal keratinocyte proliferation through p53 suppression while simultaneously controlling differentiation through a p53-independent mechanism [96].

**Fig. 5.**
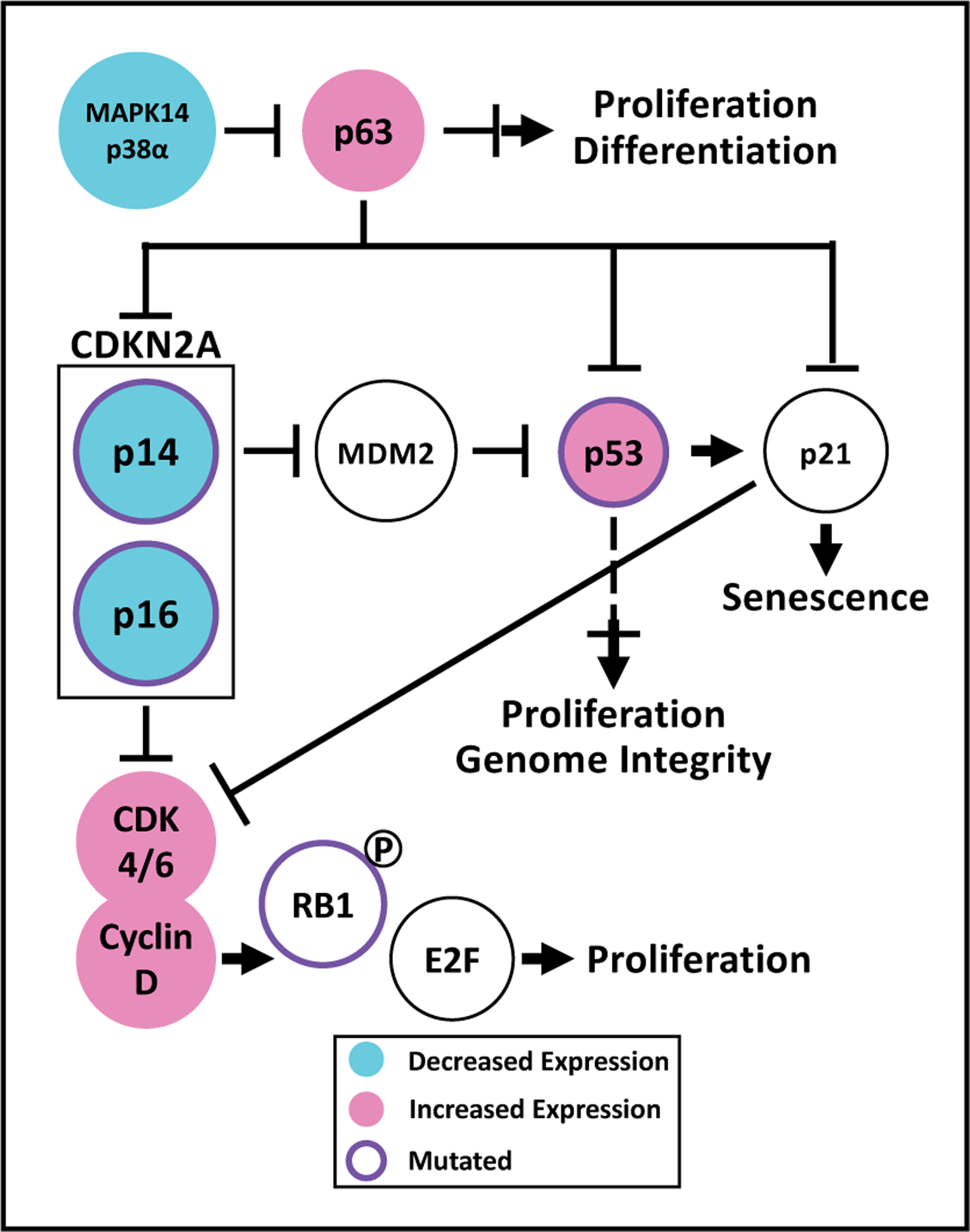
Aberrations affecting cell cycle regulators in cSCC. This figure depicts the described interactions between cell cycle regulators and the aberrations described in patients’ cSCC tumors. Decreased in cSCC, p38α normally suppresses the oncogene p63, which is recurrently amplified in cSCC. p63 inhibits p53 and p21 to promote progenitor keratinocyte proliferation and represses expression of CDKN2A, which produces the tumor suppressor proteins, p14 and p16. CDKN2A is recurrently mutated and deleted in cSCC. p16 and p21 suppress the activity of the CDK4/6-cyclin D complex. Expression of the cyclin D complex is increased in cSCC, phosphorylating RB and releasing the inhibition of the pro-proliferative E2F transcription factors. Meanwhile p14 normally inhibits MDM2, which is the primary inhibitor of p53. p53 mutation occurs frequently and early during cSCC carcinogenesis. Mutant p53 protein resists degradation, accumulates, and drives malignancy.

Sequencing of cSCC tumors reveals recurrent p63 amplification [71], but not mutation (Fig. 5). Consistent with sequencing, IHC demonstrates significantly increased p63 protein in cSCC [97]. *In vivo* experiments using mouse models demonstrate that ΔNp63α overexpression promotes cancer progression. p63 overexpression impairs apoptosis after UV exposure [98] and induces epidermal hyperplasia without spontaneous tumor formation. During DMBA/TPA tumor induction, p63 overexpression accelerates and increases tumor formation [99]. Consistently, factors enhancing p63 stability also promote carcinogenesis. STXBP4 opposes p63 degradation mediated by the anaphase promoting complex. In human cSCC, high STXBP4 expression significantly correlates with high ΔNp63α expression and poor disease staging. In a mouse xenograft model, overexpression of p63, degradation-resistant p63, or STXBP4 increases tumor volume [100]. Similar to the anaphase promoting complex, p38α (MAPK14) marks p63 for proteasomal destruction. While AK retain some p38α, it is dramatically reduced in cSCC (Fig. 5). Mice with epidermal p38α knockout express greater p63 protein causing increased keratinocyte proliferation and migration. During DMBA/TPA tumor induction, p38α knockout mice develop more papillomas than WT mice with unique malignant features including: full-thickness proliferation, impaired differentiation, vascularization, and myeloid infiltration [101]. Interestingly, ΔNp63α induces expression of MKP3, a phosphatase that represses p38 activity. ΔNp63α knockdown increases p38 phosphorylation, leading to p21 phosphorylation, Rb dephosphorylation, and G1 arrest [102]. These studies demonstrate that mechanisms increasing the amount of p63 protein can promote cSCC.

ΔNp63α represses CDKN2A, which produces the two tumor suppressors, p16 (p16^INK4a^) and p14 (p14^Arf^). In cultured keratinocytes, ΔNp63α overexpression bypasses passage-induced senescence through p14/p16 inhibition [99, 103]. p16 loss selectively immortalizes keratinocytes by increasing telomere length and maintaining expression of hyperphosphorylated Rb. Despite immortalization, the cells can stratify and differentiate with prominent hyperproliferation [104]. Further, p63 overexpression and p14 knockout can cooperate in carcinogenesis. p63 overexpression increases expression of basal keratinocyte markers, but these cells can initiate differentiation yet continue to proliferate abnormally [56]. Upon xenograft, ΔNp63α overexpressing or p14 knockout keratinocytes form poorly differentiated, necrotic tumors [56, 103] while combining ΔNp63α overexpression with p14 knockout significantly increases tumor volume [103].

In contrast to ΔNp63α, the TAp63 isoform functions as a tumor suppressor. TAp63 is not normally detectable in epidermis but is induced upon stress [105]. A component of the Fanconi anemia pathway, FANCD2 stimulates TAp63 expression in response to DNA damage [106]. TAp63 knockout induces DNA damage, including chromosome aberrations and aneuploidy. Knockout mice chronically exposed to UVR develop more primary cSCC tumors and subsequent lung metastases [107]. Finally, TAp63 is required for oncogene-induced senescence [106].

### p73

The other p53 paralog, p73 is expressed in basal keratinocytes but is dispensable for epidermal development and differentiation. Knockout mice demonstrate impaired wound healing due to increased DNA damage and decreased proliferation at the wound edge [108]. In various cancers, p73 on chromosome 1p36 is frequently lost, but rarely mutated [109]. Cultured cells treated with DMBA lose p73 protein during malignant conversion. Additionally, TAp73 knockdown keratinocytes resist radiation-induced apoptosis and senescence and form malignant tumors upon xenograft. Restoration of TAp73, but not ΔNp73, reduces tumor formation [110]. Compound p53^+/−^ p73^+/−^ heterozygous mice spontaneously develop cSCC that readily metastasize [111]. Overall, these limited results suggest that p73, especially TAp73, functions as an epidermal tumor suppressor of cSCC.

### Dysregulation of the G1/S Checkpoint

Uncontrolled proliferation is a hallmark of cancer. The “restriction point,” also known as the R point, serves as a critical checkpoint for cells to incorporate environmental cues before entering the cell cycle. R point regulation is often disrupted in cancer. Regulation of retinoblastoma (Rb) phosphorylation controls the R point, before G1 to S transition. The cyclin D1-CDK4/6 complex initiates Rb phosphorylation and the release of pro-proliferative E2F transcription factors. In cSCC, CDK4/6, cyclin D1, and Rb are frequently dysregulated.

Overexpression of cyclin D1 in keratinocytes increases proliferation, even in the presence of increased calcium, which normally induces terminal differentiation. While normal skin is typically negative for cyclin D1 by IHC, BD and cSCC (70–87% positive) demonstrate progressively increasing positivity, which correlates to sun exposure and worsening dysplasia [112–115]. At least 20% of cSCC exhibit gain of chromosome 11q13, where cyclin D1 resides (Fig. 5) [116]. In mice, oncogenic H-Ras increases cyclin D1 expression, cyclin D1-CDK4 association, and Rb phosphorylation. Cyclin D1 knockout delayed tumor formation and reduced tumor size and multiplicity upon TPA/DMBA treatment [117]. DMBA treatment induces faster and increased papilloma formation in mice overexpressing cyclin D1 [118]. Thus, increased cyclin D1 expression is a recurrent feature associated with cSCC.

Like cyclin D1, CDK4/6 is upregulated in cSCC tumors (Fig. 5), in part from the reduction of the transcription factor NFIB, which normally represses their expression. NFIB knockdown induces larger tumors with increased CDK4/6 and pRb [119, 120]. The CDK4/6 inhibitor, rafoxanide, decreases total and phosphorylated CDK4/6, cyclin D1, and pRB thus suppressing the G1/S transition and reducing cSCC tumor volume in mice [121].

Downstream, RB1 is mutated in 11.5 to 18.2% of cSCC tumors (Fig. 5) with an enrichment of nonsense and splice site mutations [44, 122]. While Rb is dispensable for epidermal development, knockout induces a lifetime of epidermal hyperplasia without spontaneous tumor formation despite its well-known tumor suppressor function. Mice with epidermal knockout of both Rb and E2F1 exhibit enhanced hyperplasia and disordered differentiation and develop wound-like cSCC tumors, indicating that E2F1 loss promotes cSCC in the absence of Rb [123]. Epidermis requires intact Rb and E2F1 function to maintain homeostasis and to suppress cSCC formation.

CDKN2A produces p14 and p16, two distinct tumor suppressors through alternative splicing. p16 binds CDK4/6 to inhibit Rb phosphorylation while p14 stabilizes p53 by inhibiting MDM2. cSCC demonstrates pervasive CDKN2A inactivation through a variety of mechanisms, including recurrent loss of chromosome 9p21 [71, 122], promoter hypermethylation [124], and mutation affecting 23% [7] to 61.5% of tumors (Fig. 5) [44, 55]. These alterations correlate with disease-specific death and shorter survival [91]. In mice, CDKN2A knockout enhances UV-induced tumor formation [125]. Given their importance, mutant p14/16 are attractive targets, but are considered difficult to drug. Epigenetic alterations to CDKN2A are being targeted by drugs such as 5-Aza-2’-dexoycytidine, approved for hematological malignancies, to induce CDKN2A promoter demethylation [126]. However, this approach cannot address CDKN2A mutation.

### MYC

MYC is essential for epidermal homeostasis and wound healing. Embryonic c-MYC ablation reduces basal cellularity and induces premature differentiation causing skin tightness that limits movement and slow-healing wounds in adult mice [127]. Mice with conditional, epidermal c-MYC ablation resist Ras-induced oncogenesis [128]. In contrast, sustained suprabasal expression of c-MYC induces proliferation, hyperplasia, papilloma formation, and angiogenesis [129], which are key processes associated with cancer progression.

AK and cSCC frequently demonstrate MYC positivity by IHC, which associates with poor tumor differentiation [130, 131]. Increased MYC expression in cSCC results from genomic amplification [71] and post-translational modifications that increase MYC protein stability. Serine 62 phosphorylation, which increases stability, is increased, and threonine 58 phosphorylation, which targets MYC for degradation, is consistently reduced in patient cSCC tumors. During DMBA/TPA treatment, epidermal expression of MYC^T58A^ (a degradation resistant mutant) decreases tumor latency and increases malignant transformation and metastasis compared to MYC overexpression. MYC overexpression actually decreases the epidermal stem cell population while MYC^T58A^ increases stem cell number and drives the stemness program [132]. As in other cancers, MYC functions as a potent oncogene in epidermal tissue.

### Epigenetic regulators

Epigenetic regulators, including histone/DNA modifiers and ATP-dependent chromatin remodelers, play critical roles in gene regulation. For an overview of this topic, please refer to Audia et al. [133]. These regulators and the epigenetic landscape are commonly dysregulated in cancer, including cSCC. Here, we present the dysregulation of methylation, acetylation, and chromatin remodelers described in cSCC.

#### DNA Methylation.

DNA methylation dynamics in normal epidermis are altered with age and sun exposure. Analysis of sun exposed and protected skin from young and old individuals reveals that dermal methylation remains unaffected while the epidermis experiences dramatic methylation changes influenced by both aging and sun exposure. Widespread heterochromatin hypomethylation in aged and exposed samples affects the same sites hypomethylated in cSCC [134]. In a survey of sun-exposed/protected skin samples, methylation readers and writers were nearly universally dysregulated in sun exposed skin with increased DNA methyltransferases (DNMT1 and DNMT3B) and decreased DNMT3A and the demethylases TET1, TET2, and TET3 [135]. Analysis of AK, primary cSCC, and metastatic cSCC reveals that CpG methylation decreases during the transition from AK to cSCC and then increases in metastatic cSCC. Gene bodies gain methylation while promoters preferentially lose methylation [136]. When the epidermal portion of cSCC and normal skin is separated and subjected to bisulfate sequencing, promoter and gene bodies demonstrate hypermethylation while repetitive sequences upstream of and within gene bodies are hypomethylated [135]. The methylation profiles of normal skin are relatively homogeneous when compared to the heterogenous methylation displayed by AK and cSCC. However, AK and cSCC consistently cluster into two groups, suggesting that some lesions arise from more progenitor-like precursors while others arise from more differentiated cells [137].

#### Histone Methylation.

The enzymes controlling histone methylation are also frequently dysregulated in cSCC. KDM1A is a repressive histone demethylase that removes H3K4me. KMD1A is overexpressed [138] while the opposing lysine methyltransferases KMT2C and KMT2D are mutated in 39% and 69.2% of cSCC respectively [72]. KMT2C mutation significantly associates with invasion and decreased survival [72]. These modifiers may represent therapeutic targets since KDM1 inhibition induces differentiation and reduces dermal invasion in mice [138]. The polycomb repressive complexes also write histone methylation. RING1B of complex 1 and EZH2 of complex 2 are increased in metastasizing versus non-metastasizing cSCC [139]. EHMT2, an H3 lysine methyltransferase, is increased in cancer. EHMT2 deletion in mice increases chromatin accessibility at regulatory sites with minimal gene expression changes. However, deletion induces replicative stress and genomic instability. While knockout delays cSCC induction, the tumors are aggressive. Depletion in established tumors causes regression, but regressed lesions relapse into more aggressive, poorly differentiated tumors [140]. Thus, this EHMT2 study provides a cautionary tale about the unexpected outcomes of genome-wide modification.

#### Acetylation.

The evidence regarding the role of CREBBP and p300 in cSCC is limited and conflicting. Both are lysine acetyltransferases that function as transcriptional activators. At least one functional allele of each is required for epidermal development. CREBBP mutation affects ~30% of cSCC tumors. In contrast to other cancers, which disclose indel mutations, primary cSCC tumors harbor CREBBP missense mutations. Yet, cSCC lymph node metastases acquire CREBBP indels, suggesting loss of function promotes tumor progression [141]. In contrast, increased expression of p300 significantly associates with lymph node metastasis, advanced tumor stage, and poor survival [142, 143]. In mice, heterozygous loss of EP300 or CREBBP cooperates with HRAS^S35^ to induce postnatal epidermal thickening and spontaneous papilloma formation due to EGFR hyperactivation [144].

Preclinical testing in murine cSCC models suggests that HDAC inhibitors could be a promising cSCC treatment strategy. HDACs are histone deacetylases, and HDAC inhibitors are already approved for a number of hematological malignancies. HDAC3 is increased in cSCC. The ginseng derived compound 20(R)-Rg3 decreases HDAC3 protein. Either drug treatment or HDAC3 knockdown reduces EMT and migration [145]. Likewise, the compound MS-275 inhibits class I HDACs leading to upregulation of H3K9ac. Low-dose treatment inhibits the proliferation of human cSCC cell lines and significantly reduces the size of UV-induced tumors [146]. Yet another inhibitor, vorinostat reduces HDAC1/2/3/7 activity and increases histone and non-histone acetylation. Treatment reduces proliferation, increases apoptosis, and induces large areas of necrosis in cSCC xenografts [147]. Targeting cSCC acetylation appears promising, and FDA-approved drugs can be repurposed more quickly than new drugs can be developed.

#### The BAF Complex.

The BAF family of chromatin remodeling complexes regulate DNA accessibility for transcription. BAF genes are highly mutated in cancer and can function either as an oncogene or tumor suppressor in a tissue dependent manner [148]. The ATPase subunits, BRM and BRG1 are significantly and consistently reduced in cSCC tumors by IHC [149]. BRM knockout induces severe epidermal hyperplasia and abnormal proliferation of the suprabasal keratinocytes in mice upon UV radiation [150]. The BRM null cells enter G1 arrest but escape prematurely and accumulate DNA damage without appropriate DNA repair or apoptosis [151]. Another BAF subunit, ACTL6A, suppresses the differentiation program in progenitors by sequestering the complex from promoters of genes required for differentiation. Consequently, ACTL6A loss induces cell cycle exit and epidermal hypoplasia [152]. cSCC exhibits intense ACTL6A overexpression, and 20% of tumors harbor ACTL6A amplifications. Unlike in normal epidermis, ACTL6A and p63 are co-expressed in cSCC and robustly interact to promote the progenitor transcriptional program. ACTL6A knockdown inhibits proliferation, invasion, and xenograft tumor growth while overexpression promotes rapid growth [153, 154]. Within the same complex, BRM/BRG1 and ACTL6A play opposing roles in cSCC formation. Chromatin remodelers remain understudied in epidermal homeostasis and cSCC despite containing potentially druggable ATPase domains.

## Alterations to Non-coding RNAs

An emerging literature indicates that non-coding RNAs (ncRNAs) play crucial roles in regulating epidermal differentiation, wound healing, and UV response and function as both oncogenes and suppressors in cSCC [155]. The ncRNAs characterized in cSCC include long non-coding RNAs (lncRNAs), microRNAs and circular RNAs, summarized in Table 1. These ncRNAs can influence gene expression in both the nuclear and cytoplasmic compartments. However, relatively few of these ncRNAs have been thoroughly characterized in normal epidermal development or homeostasis.

lncRNAs are transcripts >200 nucleotides with diverse regulatory functions including: scaffolding molecular complexes, guiding the binding of chromatin remodelers and transcription factors, modulating mRNA processing, and inhibiting (sponging) microRNAs [156]. Comparing cSCC to healthy skin by RNA sequencing reveals differential expression of 908 lncRNAs in cSCC with a bias towards downregulation [157, 158]. circRNAs are subset of lncRNAs with covalently closed ends [156], which share the downregulated trend (53/55 decreased) in cSCC. Additionally, the negative regulator of circRNA biogenesis, ADAR, is upregulated while positive regulators, MBNL and ESRP1, are downregulated [158]. All four of the circRNAs described in cSCC sponge microRNAs with tumor suppressor properties.

microRNAs are ~22 nucleotide RNAs that inhibit the translation of >60% of all mRNAs. Many of the microRNAs described in cSCC converge on the p63 and MAPK pathways, which are critical for epidermal homeostasis and carcinogenesis. microRNAs show therapeutic promise in preclinical models. For example, miR-634 synergizes with EGFR inhibition to reduce tumor growth in mice [159] while restoring miR-3619–5p expression in cisplatin resistant cell lines improves chemotherapy efficacy [160]. microRNA-based therapies are currently being developed and tested in clinical trials. Therapy is complicated by the potential for off-target effects and the myriad technical details regarding delivery of these fragile RNAs or their antisense inhibitors to tumors. For a summary of systemic delivery options, see Rupaimoole et al. [161] and Ross [162] for topical delivery.

## Alterations to the Extracellular Environment

**Fig. 6.**
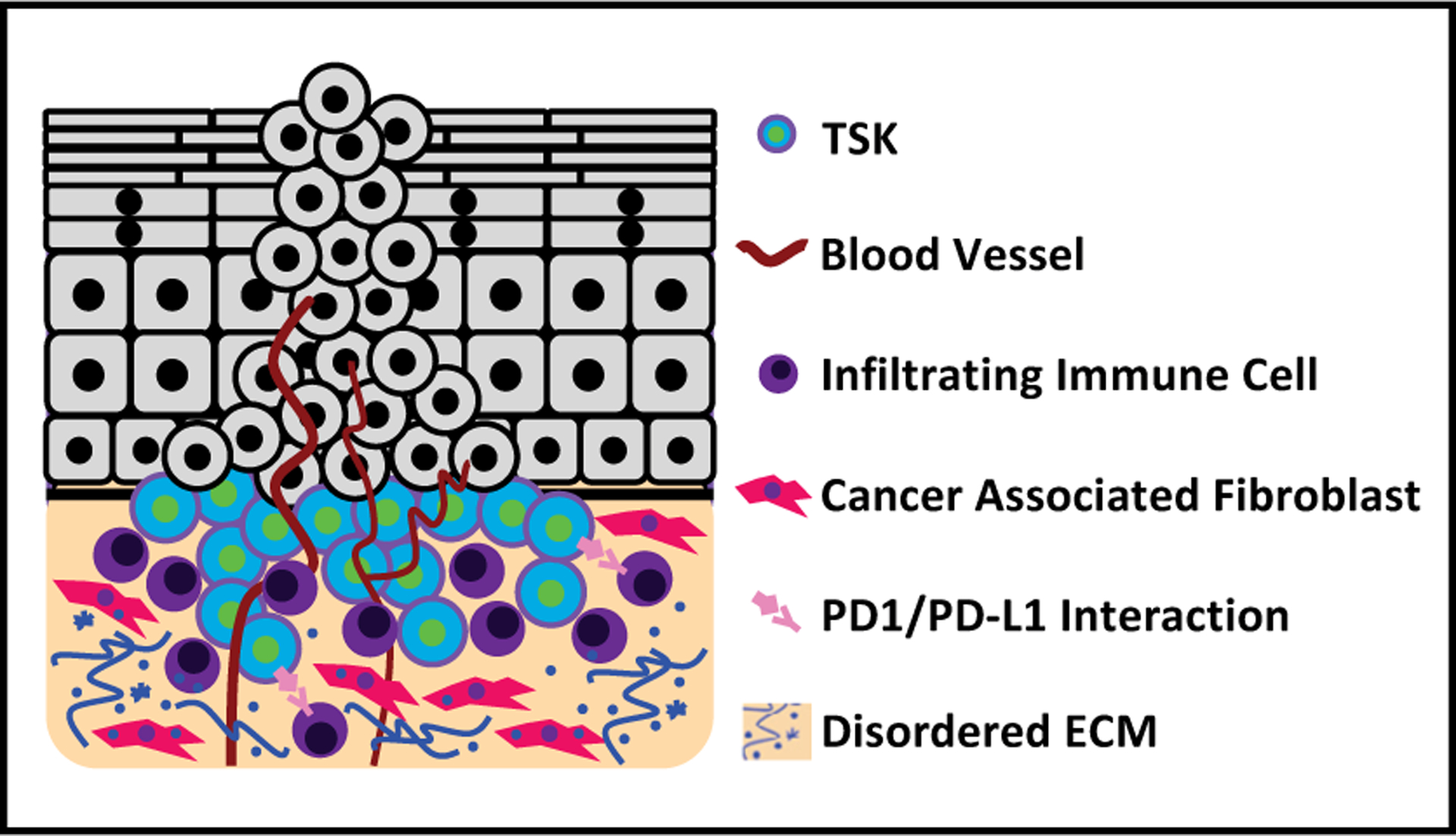
The extracellular environment in cSCC. At the tumor’s invasive front, cSCC cells disrupt the basement membrane and invade the dermis. In response, dermal fibroblasts become cancer-associated fibroblasts (CAFs) and promote cSCC progression. Immune cells migrate to the tumor, where they typically surround, but do not invade the tumor. cSCC tumors and infiltrating immune cells demonstrate PD1 and PD-L1 positivity, which disrupts immune mediated killing and surveillance. Both CAFs and infiltrating immune cells secrete ECM components and remodeling enzymes, creating a microenvironment that promotes invasion and angiogenesis.

cSCC cells at the invasive front interact with the various components of the dermis, including: dermal fibroblasts, immune cells, and the extracellular matrix (ECM). Dermal fibroblasts secrete and organize the ECM, which is primarily composed of proteins and carbohydrates that orient and anchor cells and participate in signaling. Fibroblasts and immune cells secrete enzymes that digest the ECM, which contributes to angiogenesis and cSCC invasion. Finally, immune surveillance is perturbed. This section provides an overview of these alterations (Fig. 6) and their contribution to cSCC progression.

### Dermal Fibroblasts

cSCC alters fibroblast gene expression and behavior, producing a population of cancer associated fibroblasts (CAFs). These CAFs cross talk with keratinocytes to promote cSCC progression. Ras-mutant HaCaT cells cannot invade collagen gels unless fibroblasts are present [163]. Further, cSCC cell lines cultured with primary CAFs in organotypic culture detach from and invade the dermis. Co-culture with CAFs impairs epidermal differentiation, but paradoxically reduces cSCC proliferation compared to co-culture with normal fibroblasts [164]. In mice, fibroblast proliferation increases during DMBA/TPA induction. Fibroblast depletion delays papilloma formation, reduces malignant transformation by half, and reduces macrophage tumor infiltration [165].

CAFs isolated from patient tumors demonstrate dysregulation of key regulatory pathways. CAFs associated with AK and cSCC demonstrate downregulation of ATF3, a transcription factor transiently induced by UV radiation in normal fibroblasts. ATF3 deletion in fibroblasts promotes the secretion of growth factors, cytokines, and matrix modulating enzymes. Xenograft of ATF3 depleted fibroblasts with cSCC cells increases dysplasia and induces formation of invasive, ulcerated cSCC with impaired differentiation [166]. CAFs also demonstrate altered WNT and NOTCH signaling. About 25% of cSCC disclose nuclear beta-catenin in CAFs instead of the membranous staining observed in normal dermis. These CAFs are highly susceptible to WNT induction, which induces secretion of cytokines and matrix components promoting keratinocyte proliferation [167]. Further, CAFs isolated from patient cSCC tumors recurrently disclose NOTCH1 amplification. Silencing NOTCH1 partially normalizes CAF gene expression, impairs cSCC cell growth in co-culture, and reduces tumor size, macrophage infiltration, and angiogenesis in xenograft [168]. Additionally, CAFs exhibit dysregulated FGF and TGFβ signaling, which oppositely regulate the transcription factor ETV1. Abnormal signaling increases growth factor and cytokine production, ECM remodeling, macrophage recruitment, and promotes tumor growth [169]. Both epidermal keratinocytes and dermal fibroblasts accumulate mutations and signaling alterations which drive abnormal cellular interactions and cSCC progression.

### Tumor Infiltrating Immune Cells

Homeostatic skin hosts a battery of resident immune cells [170]. During malignant transformation, additional immune cells are recruited, where they contribute to cancer progression. For example, DMBA/TPA induction recruits neutrophils. In papillomas, neutrophils promote ECM remodeling, angiogenesis, and metastasis while neutrophils in tumors alter metabolism and suppress immune response [171]. Xeroderma pigmentosum, caused by a deficiency in DNA repair, promotes childhood cSCC development. Fibroblasts from both xeroderma pigmentosum patients and sporadic cSCC lose the receptor CLEC2A, which activates natural killer cells. In organotypic culture containing cSCC cell lines, normal fibroblasts, and natural killer cells, introduction of anti-CLEC2A antibody promotes invasion to the same extent as culture containing xeroderma pigmentosum fibroblasts [172]. Compounding natural killer inactivation, greater than 75% of spontaneous cSCC tumors express another cell-surface receptor LLT1, which inhibits natural killer cell. Expression of LLT1 associates with increased tumor thickness, nodal metastasis, and death [173]. Although neutrophils and natural killer cells are recruited to cSCC tumors, their normal anti-tumor activity is subverted or inhibited.

Although macrophages are the most abundant immune cell in homeostatic skin, premalignant and cSCC lesions progressively recruit even more macrophages [174]. These tumor associated macrophages (TAMs) primarily surround rather than infiltrate cSCC tumors and become dysfunctional. For example, normal macrophages can be broadly classified as either M1 (pro-inflammatory) or M2 (regulatory, wound healing) but many TAMs exhibit abnormal M1/M2 bi-activation [175]. Additionally, TAMs secrete VEGFC, MMP9, and MMP11 which collectively promote new vessel formation and metastasis [175, 176]. Because of their abundance and dysfunction, TAM depletion may be a viable strategy to treat cSCC. In a murine model, macrophage depletion inhibits tumor growth, decreases vessel penetration into tumor, and reduces invasion [177]. Likewise, a phase III trial demonstrates that oral nicotinamide dramatically reduces TAMs, which may contribute to its ability to prevent new tumor formation [178].

T cell dysfunction is a key feature of cSCC. T cells accumulate in the peritumor environment and express markers of exhaustion, which is the inability to effectively respond to antigen stimulation [13]. Dendritic cells process and present antigens to T cells. Like other immune cells, dendritic cells are increased in the peritumoral area. Yet, dendritic cells isolated from cSCC are unable to stimulate T cells compared to dendritic cells isolated from normal human skin [179]. In fact, cSCC-derived dendritic cells can even suppress T cell proliferation in ex vivo culture [180]. However, some cSCC associated immune cells increase T cell activation. Langerhans cells are epidermis-resident macrophages with dendritic properties. cSCCassociated Langerhans cells can induce greater proliferation of CD4+ and CD8+ T cells than Langerhans cells from normal skin. This suggests that utilizing tumor Langerhans cells in therapy could be a promising strategy [180].

Further contributing to T cell dysfunction, is the dysregulation of the PD-1/PD-L1 axis in cSCC. In healthy tissue, the programmed death-1 receptor (PD-1) and its ligands, PD-L1 and PD-L2, protect tissue by preventing excessive T cell activity. Tumor cells activate PD-L1 expression to escape T cell mediated killing. Additionally, tumor infiltrating immune cells also express PD-L1. This induces an immune suppressive phenotype in regulatory T cells, inhibits naïve CD8^+^ T cell activation, and prevents reactivation of exhausted CD8^+^ T cells, the cells that likely recognize tumor neoantigens [181].

The presence of PD-L1^+^ cells predicts lymph node metastasis and poor differentiation [182, 183]. However, disagreement remains in defining what constitutes PD-L1 positivity with different studies setting widely varying thresholds and examining different cell types. 26% of primary cSCC tumors from immunocompetent patients exhibit PD-L1^+^ tumor cells, 60% of cSCC harbor PD-L1^+^ infiltrating immune cells, and 81% have PD-1^+^/CD8^+^ T cells [184]. Another study showed that 41% of cSCC tumors harbor PD-L1^+^ dendritic cells and 61% contain PD-L2^+^ dendritic cells, which correlates with increased tumor size, and counterintuitively well-differentiated status [185]. During DMBA/TPA induction, PD-L1^+^ neutrophils infiltrate tumors and inhibit CD8^+^ T cells. Neutrophil depletion delays tumor growth [171]. Positivity persists in metastatic cSCC, where ~33% of tumor cells are PD-L1^+^ and correlates with intra- and peritumoral CD8^+^ T cell infiltration and poor differentiation of the primary tumor [186, 187]. Together these results suggest the PD-1 and PD-L1 positivity are prevalent in cSCC and that PD-L1 positivity implies poorer disease outcomes. In contrast, the role of PD-L2 has not been satisfactorily elucidated.

In 2018, the FDA approved cemiplimab, a PD-1 antibody, as the first treatment for advanced cSCC. The phase I trial enrolled 26 patients and achieved an overall response rate (ORR) of 50% while the phase II trial enrolled 193 immunocompetent patients and achieved ORR of 41.1–49.1% with durable response. Interestingly, responders had a higher mutational burden of 53.2 to 74.2 per Mb compared to 13.7 to 28.7 per Mb for non-responders, suggesting that mutational burden may predict patient response [188]. Likewise, pembrolizumab, another PD-1 antibody achieved an ORR of 34.2% in patients with unresectable cSCC [189]. cSCC tumors were classified as PD-L1 positive or negative by IHC. The ORR for PD-L1^+^ patients was 55% versus 17% for PD-L1^−^ patients [190]. Currently, clinical trials are enrolling cSCC patients to test nivolumab (PD-1 antibody), IBI318 (PD-1/PD-L1 bispecific antibody), and avelumab, atezolizumab, and cosibelimab (PD-L1 antibodies). Immune checkpoint blockade will likely become standard of care for cSCC, which highlights the need for standardized biomarkers that predict patient response. Furthermore, managing toxicity remains critical, especially for organ transplant patients who risk graft rejection [191].

### Extracellular Matrix

The extracellular matrix (ECM) is the non-cellular component of tissues which provides physical and biochemical support to the cells. Both the structural proteins and the enzymes that remodel them are dysregulated in cSCC. The chief structural ECM proteins are collagens. COL7A1 mutation causes recessive dystrophic epidermolysis bullosa (RDEB). Patients experience chronic blisters, fibrosis, inflammation, and early onset cSCC that readily metastasizes. The loss of COL7A1 induces disorganization of collagen fibers and fibroblast activation similar to that observed in spontaneous cSCC. In RDEB, the collagen cross-linker LOX, integrin β1, and the kinase FAK also increase which stiffen the ECM. This stiffening mechanically induces the pro-migration and pro-survival integrin-AKT signaling axis [192, 193]. cSCC tumors also demonstrate suprabasal stiffening. This combined with proliferation-driven tissue deformation and dysregulated remodeling of the basement membrane promotes invasive properties [194].

Integrins are heterodimeric cell surface receptors that mediate signaling to regulate cellular adhesion, migration, proliferation, and survival. Integrins are extensively dysregulated in cancer [195]. In the Ras-induced cSCC model, the stroma demonstrates rapid changes which promote angiogenesis followed by dysregulation of ECM and adhesion genes while antigen presentation is progressively repressed. Integrins (ITGB1, ITGB4, ITGA3, IGBA5, and ITGA6) govern this network of dysregulated stroma genes. ITGB1 increases during malignant progression, and antibody treatment reduces tumor growth and improves the epidermal-dermal boundary in a xenograft model [196]. Another integrin, ITGB4 is a component of the hemidesmosome, an anchor between keratinocytes and the ECM. ITGB4 undergoes increased N-glycosylation in cSCC. Interrupting this glycosylation reduces tumorigenesis [197]. Additionally, CAFs produce an integrin ligand, periostin. Although absent in normal ECM, periostin is prominently expressed in high risk cSCC and associates with larger tumors, invasion, and poor differentiation [198].

Laminins are heterotrimeric glycoproteins composed of α, β, and subunits. Laminin 332 (α3 β3 γ2) is the primary functional laminin in skin, and its receptors, integrins α3β1 and α6β4, are expressed on the surface of keratinocytes. Laminin α3 is decreased in poorly differentiated cSCC tumors [199]. IHC reveals γ2 staining at the invasive edge of cSCC. Spheroid co-culture with fibroblasts induces Ras-mutant, but not wild-type, keratinocytes to produce lamin-332 and to invade collagen gels. Treatment with anti-integrin antibody prevents invasion in *in vitro* assays [200]. Knockdown of α3 or γ2 reduces keratinocyte adhesion. In xenografts, α3 or γ2 knockdown produces larger, more invasive tumors. β3 knockdown tumors are larger than control, but more differentiated and not invasive. α3 or γ2 knockdown tumors recruit more macrophages that infiltrate the tumors [199].

Matrix metalloproteases (MMP) are calcium- and zinc-dependent enzymes that degrade components of the ECM. cSCC demonstrates an overall upregulation of MMP activity, which promotes invasion and angiogenesis. Tumor cells at the invasive front overexpress several MMPs (1, 2, 3, 7, 9, 11, 13, and 14). Invasive cells, but not normal epidermis, express MMP1, 3, and 7. In contrast, MMP9 and 10 are expressed by normal basal keratinocytes, but expression increases in cSCC [201–204]. Infiltrating immune cells contribute yet more MMP8 and 9 to the tumor environment [205]. cSCC and the adjacent dermis exhibit intense MMP10 staining, which associates with poor pathology [206]. MMP2, MMP14, and TIMP2 form a trimeric, membrane-bound complex. MMP14 activates MMP2. Weak MMP14 expression occurs in normal basal cells, and is elevated at the invasive front. MMP2 expression correlates with cSCC progression [201, 203, 207]. TIMP2 inhibits MMP2 and is reduced in cSCC tumors [201]. Additionally, the surface-bound glycoprotein CD147 stimulates MMP activity and is strongly expressed by primary cSCC and metastases. CD137 expression in primary tumors promotes metastasis [208]. Given the strong upregulation, MMPs are attractive therapeutic targets. Various MMP inhibitors are under pre-clinical and clinical investigation, but this approach is fraught with problems. Early, zinc-chelating inhibitors acted non-specifically and induced serious side effects. Antibody-based therapies provide specificity but are easily degraded. To achieve anti-tumor effects rather than inadvertently promoting metastasis, target selection, treatment timing, and drug specificity require further refinement [209].

## Conclusion

cSCC represents an unmet clinical need that will continue to grow for the foreseeable future. cSCC develops from keratinocytes that accumulate a massive mutational burden, which impacts regulation of gene expression and signaling pathways. This mutational burden not only complicates the identification of important drivers of progression, but also facilitates synergy among oncogenes to accelerate cSCC progression. In addition, abnormal dermal fibroblasts and an abundance of infiltrating immune cells contribute to the cancerous phenotype by shaping the microenvironment. Understanding the complex interactions between these cellular components is crucial to fully describe cSCC dynamics and develop effective therapies. Currently, the only two approved therapies for cSCC are cetuximab, an anti-EGFR antibody that targets keratinocyte dysfunction, and cemiplimab, an anti-PD-1 antibody, that targets inappropriate immune interactions. Yet, both approved therapies would benefit from the discovery of biomarkers that robustly predict successful patient responses. Therapies specifically tested in and approved for cSCC are still needed. Fortunately, a number of promising targets have already been identified, and potential therapies are currently being developed or repurposed. Further research is required to discover novel druggable targets and biomarkers that reliably predict patient prognosis.

## Figures and Tables

**Table 1.** A summary of non-coding RNAs and their described roles as tumor suppressors or oncogenes in cSCC